# Should Spinal Anesthesia Be Used for Spine Surgery: A Case Report

**DOI:** 10.1155/cria/7810025

**Published:** 2025-04-28

**Authors:** Gabrielle A. White-Dzuro, Xiaodong Bao, Francis McGovern, Robert A. Peterfreund

**Affiliations:** ^1^Department of Anesthesia, Critical Care, and Pain Medicine, Massachusetts General Hospital, Boston, Massachusetts, USA; ^2^Department of Urology, Massachusetts General Hospital, Boston, Massachusetts, USA

**Keywords:** lumbar spine surgery, spinal anesthesia

## Abstract

A patient with lumbar spine stenosis presented for lumbar spine decompression, clearly stating a preference for spinal anesthesia (SA) over general anesthesia (GA). Surgery was performed under uneventful SA without sedation. The patient recovered from surgery quickly and without incident, requiring no postoperative opioid analgesia. What data support administering SA for lumbar spine surgery? A literature search found nine recently published reviews favoring SA over GA. These reviews cited many of the same, older, flawed, primary research reports. Although choosing SA was reasonable, our literature analysis suggests blind reliance on summary conclusions of reviews and meta-analyses may be misleading.

## 1. Introduction

Reports of spinal anesthesia (SA) for lumbar spine surgery have been published for more than 60 years [1]. Over the past decade, nine English language meta-analyses and review publications suggested advantages of SA over general anesthesia (GA) [2–10]. Five were published in the last 3 years [4–7, 10]. Touted benefits of SA include decreased operative time, estimated blood loss, postoperative nausea and vomiting, improved pain control, and increased reimbursement [2–4, 6–8, 10, 11]. However, SA has yet to be widely adopted for lumbar spine surgery. A surgeon presented for lumbar decompression, requesting SA for surgery. What data support this choice? We describe a case of SA for lumbar spine decompression and analyze recent review publications comparing SA to GA, discussing their limitations. Written Health Insurance Portability and Accountability Act (HIPAA) authorization was obtained from the patient for publication of this case report and this manuscript adheres to the applicable EQUATOR guideline.

## 2. Report of Case

A 65-year-old surgeon (180 cm, 75 kg, body mass index [BMI] 23) with a past medical history of mild hypertension and hyperlipidemia presented with lower back pain radiating into the gluteal and posterior thigh region bilaterally. Neurologic examination revealed 5/5 strength in bilateral hip flexion, hip adduction, hip abduction, knee flexion, ankle dorsiflexion, inversion, and eversion. Magnetic resonance imaging demonstrated a central disc bulge at L4-L5 with bilateral moderate to severe degenerative changes of the L4-L5 facet joints resulting in moderately severe central spinal stenosis at L4-L5. The imaging findings were felt to account for the symptoms. The patient was scheduled for an L4-L5 lumbar posterior decompression of the central canal and lateral recesses.

On the day of the procedure during the informed consent discussion for anesthesia, the patient expressed a strong preference for SA without sedation. His concerns regarding GA included potential neurocognitive effects, postoperative narcotic requirements, an airway event with prone positioning, and an upper extremity positioning injury.

With the patient in the seated position, surface landmarks were used to identify the L3-4 interspace. Fluoroscopy later identified this as the L2-3 interspace. A 25-gauge (length 90 mm) Whitacre needle (Smiths Medical ASD, Keene, NH), guided by an introducer, was inserted into the thecal sac (1 attempt) with a return of clear CSF. Bupivacaine (0.5% plain solution) was injected in divided doses (2.4 mL total) achieving a T6 sensory level and a successful SA for L4-L5 decompression. The patient positioned himself comfortably in the prone position. The SA was supplemented with a bolus of intravenous fentanyl (25 mg) and local anesthetic injected in the surgical field.

During the operation, the patient did not require hemodynamic support. After an unremarkable intraoperative course, the patient was transferred to the postanesthesia care unit (PACU). After the effects of the SA dissipated, the postoperative examination found no neurological deficits with 5/5 bilateral upper and lower extremity strength. He denied nausea, vomiting, or any headache suggesting SA complications. In the PACU, the patient received acetaminophen for 2/10 postoperative pain. He was admitted for routine overnight observation. Later in the day, he resumed a regular diet and ambulated without assistance. He was discharged on postoperative day one.

At the 3 weeks postoperative visit, examination demonstrated 5/5 strength throughout his bilateral lower extremities. His preoperative back and bilateral leg pain had completely resolved. The patient expressed satisfaction with his progress and reported return to regular activity. He resumed performing surgery 4 weeks postoperatively.

## 3. Discussion

The medically knowledgeable patient's strong preference for SA prompted an examination of English language review articles and meta-analyses comparing SA to GA for lumbar spine surgery. The anesthesia team sought data supporting the patient's preference. Publications were identified by a search of the PubMed database. The literature search found 9 relevant reports. Unfortunately, there are significant limitations to applying the conclusions of the multiple review articles in this field.

In the era of opioid-free anesthesia, decreased postoperative pain and narcotic requirements are important outcomes to consider when comparing GA and SA. Six of the nine reviews found a decrease in either early postoperative pain scores or analgesic requirement when SA was used [3–6, 8, 9]. Urick et al. was unable to provide a comment on this topic due to a lack of granularity of the data [7]. De Cassai et al. noted that while they found a difference, the quality of evidence was very low [4]. Furthermore, 4 of the 9 papers noted no difference in pain scores at 24 h [2–4, 6]. Benefits of SA for pain control thus remain uncertain.

For surgery duration, 3 of the 9 publications suggested shorter operative time with SA [5, 7, 10], while four others analyzing this outcome found no difference [2–4, 8]. The one outcome that all studies agreed on is that regional anesthesia decreases the incidence of postoperative nausea and vomiting (PONV), specifically at 24 h postoperatively [2–4, 6–10].

A difficult outcome to study is intraoperative hemodynamic stability, which appears to be defined heterogeneously. No studies found a significant difference in intraoperative hypotension based on the type of anesthesia used. Three studies described favorable hemodynamic outcomes for regional anesthesia with less hypertension and tachycardia [3, 4, 6]. An important confounder in the primary studies is the unspecified use of vasoactive medications to influence the reported hemodynamic outcome.

There is another obvious deficiency in these review publications. Seven of the nine cited review articles include both SA and epidural anesthesia (EA) in their analysis of primary data [2, 5–10]. Researchers should not equate the two; they represent distinct forms of anesthesia and should be treated as such. The intention behind the authors inclusion of EA is unclear, as they neglect to address the differences between the anesthetics and even refer to the EA as intrathecal injections [5, 7].

One must consider the strength of review papers that are all based off the same six primary studies and draw different conclusions (Table 1). The oldest primary study was published in 1996 by Jellish et al. and was included in seven out of the nine review papers [2–4, 6, 8–10, 12]. The most recent of these commonly cited research reports was published in 2014 [13, 14]. The evolution of GA over that period has been significant. With the advancement of Enhanced Recovery After Surgery (ERAS) protocols and the focus on multi-modal analgesics and PONV prophylaxis, it is unclear if a more recent study would have similar findings.

In summary, providers seeking to identify potential advantages and disadvantages of SA for lumbar spine surgery should appreciate that the multiple reviews and meta-analyses have many limitations. Consequently, recommendations for SA over GA for lumbar spine surgery are not necessarily grounded in strong evidence. Overall, providers must think critically when looking at review or meta-analyses to ensure that the studies that they include are recent and relevant. These publications and the conclusions they draw are only as good as the research that they cite.

## Figures and Tables

**Table 1:** Frequency at which primary literature is represented in the meta-analyses.

Primary literature	Meta analyses									
	De Rojas et al. [8]	Mergeay et al. [9]	Zorrilla-Vaca et al. [2]	Meng et al. [3]	De Cassai et al. [4]	Perez-Roman et al. [5]	Shui et al. [6]	Urick et al. [7]	Ghaith et al. [11]	Frequency
Jellish et al. [12]	1	1	1	1	1	0	1	0	1	7/9
Sadrolsadat et al. [15]	1	1	1	1	1	1	1	1	1	9/9
Attari et al. [16]	1	1	1	1	1	1	1	0	1	8/9
Kara et al. [17]	0	0	1	1	1	0	1	0	1	5/9
Kahveci et al. [14]	0	1	1	1	1	0	1	1	0	6/9
Vural and Yorukoglu [13]	0	0	1	1	1	0	1	0	0	4/9

## Data Availability

The data supporting this case report are available upon reasonable request from the corresponding author. Due to patient confidentiality and ethical considerations, personal identifiers and sensitive information are not included in the publicly available data.

## Nomenclature

SA: Spinal anesthesia
GA: General anesthesia
HIPPA: Health Insurance Portability and Accountability Act
BMI: Body mass index
PACU: Postanesthesia care unit
PONV: Postoperative nausea and vomiting
EA: Epidural anesthesia
ERAS: Enhanced Recovery After Surgery
